# Dynamic Proteomics of Human Protein Level and Localization across the Cell Cycle

**DOI:** 10.1371/journal.pone.0048722

**Published:** 2012-11-07

**Authors:** Shlomit Farkash-Amar, Eran Eden, Ariel Cohen, Naama Geva-Zatorsky, Lydia Cohen, Ron Milo, Alex Sigal, Tamar Danon, Uri Alon

**Affiliations:** Department of Molecular Cell Biology, Weizmann Institute of Science, Rehovot, Israel; Virginia Tech, United States of America

## Abstract

Regulation of proteins across the cell cycle is a basic process in cell biology. It has been difficult to study this globally in human cells due to lack of methods to accurately follow protein levels and localizations over time. Estimates based on global mRNA measurements suggest that only a few percent of human genes have cell-cycle dependent mRNA levels. Here, we used dynamic proteomics to study the cell-cycle dependence of proteins. We used 495 clones of a human cell line, each with a different protein tagged fluorescently at its endogenous locus. Protein level and localization was quantified in individual cells over 24h of growth using time-lapse microscopy. Instead of standard chemical or mechanical methods for cell synchronization, we employed in-silico synchronization to place protein levels and localization on a time axis between two cell divisions. This non-perturbative synchronization approach, together with the high accuracy of the measurements, allowed a sensitive assay of cell-cycle dependence. We further developed a computational approach that uses texture features to evaluate changes in protein localizations. We find that 40% of the proteins showed cell cycle dependence, of which 11% showed changes in protein level and 35% in localization. This suggests that a broader range of cell-cycle dependent proteins exists in human cells than was previously appreciated. Most of the cell-cycle dependent proteins exhibit changes in cellular localization. Such changes can be a useful tool in the regulation of the cell-cycle being fast and efficient.

## Introduction

It is of interest to understand the regulation of proteins across the cell cycle – a fundamental process in cell physiology in both health and disease. Proteins can be regulated across the cycle by means of chemical modification and binding. They can also be regulated by changes in their level and localization in a cell-cycle dependent manner.

Previous studies focused on individual proteins, and discovered mechanisms that change protein levels and localization in a cell-cycle dependent manner. Global analyses have mostly used mRNA measurements, due to the availability of high throughput methods such as microarrays. Fraction of cell-cycle dependent genes have been found to range from 6% ([1] ) to 10% in budding yeast (800 genes, [2] ) and 6% in fission yeast (407 genes, [3]). Surprisingly, in human cells, only about 1–3% of messages are cell-cycle dependent [4]–[7].

Studies on the protein level are much more difficult due to current limitations of technology, especially in human cells. Recent studies [8]–[10] used time-lapse fluorescence microscopy to study the cell-cycle dynamics of yeast proteins.

One method, dynamic proteomics [11]–[13], is suited to study cell-cycle dependence of proteins in human cells. Dynamic proteomics uses a library of cell clones, each with a different protein tagged fluorescently at its endogenous chromosomal locus, with endogenous regulation [14]. Proteins in individual cells are followed at high resolution using time-lapse movies and automated image analysis. A dynamic proteomics study on 20 nuclear proteins found that 40% of the proteins showed cell-cycle dependent changes in level [15]. The study used in-silico synchronization, a method that places data on a time axis between two cell divisions based on the fact that cell divisions can be identified automatically from the movies. Use of in-silico synchronization avoided the deleterious effects of standard methods of cell synchronization using chemicals or starvation. The study of Sigal et al suggests that the prevalence of cell-cycle dependence of the protein level might be much higher than found on the mRNA level. It is of interest to test this on more proteins, especially on non-nuclear proteins, and to study protein localization in addition to protein level.

Here, we extend the dynamic proteomics approach to study the cell-cycle behavior of 495 proteins with diverse roles and localizations, and to search for cell-cycle dependent changes in both protein level and localization. We find that about 40% of proteins tested show cell cycle dependent changes in level and/or localization. Localization changes were more prevalent than cell-cycle changes in level (about 11% of proteins had cell-cycle dependent changes in level). This suggests that cell-cycle control at the level of proteins is more widespread than previously known and proposes new candidates for this specialized control.

## Results

### Time-lapse Movies of 495 Unique Proteins were Analyzed and In-silico Synchronized

**Figure 1:**
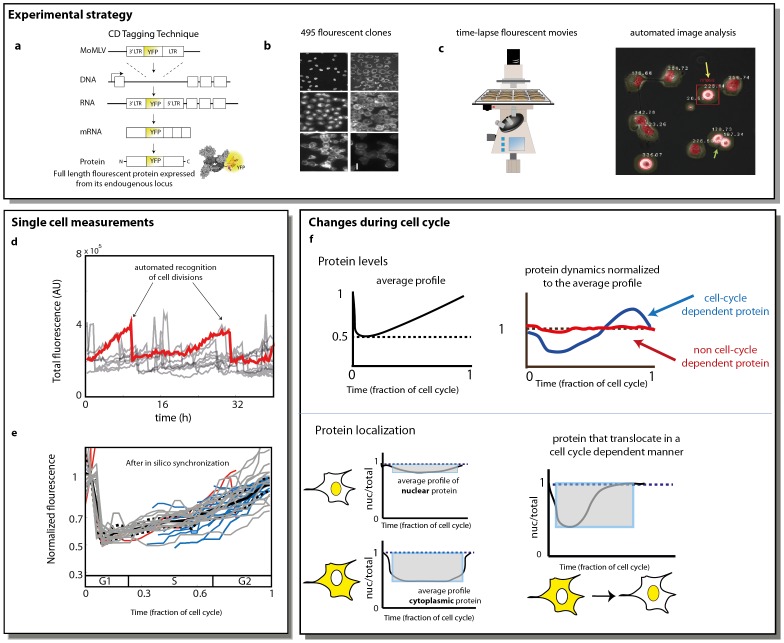
Schematic overview of dynamic proteomics for exploring cell cycle dependent changes in level and localization. (A) CD tagging was used to insert YFP as an exon into the introns of genes on the chromosome of a human cell line clone (H1299), resulting in a full length protein fused to YFP expressed from its endogenous locus. (B) A panel of 6 representative clones with different tagged proteins from the LARC library (C) Time-lapse microscopy and automated image analysis allow capturing proteins levels and localizations in individual cells over time. Yellow arrow indicates a cell in mitosis, green arrow indicates cells post mitosis. (D) Fluorescence traces of individual cells over a 40 hours movie (tagged protein is DDX5). Sharp decreases are at division events (E) In silico synchronization is done by plotting cell dynamics on a time axis which indicates time from previous or next division. Time is divided by mean cell cycle duration, to provide fraction of cell cycle elapsed. G, S and G2 phases are estimated from Sigal [15]. Grey lines- cells with two mitosis events in the movie, blue, red lines: cells with one mitotic event. The fluorescence level is normalized to the maximal level before cell division. (F) In silico synchronized dynamics are used to examine cell-cycle dependence on levels and localizations. On the top panel, Protein profile (blue) that is significantly different from the average profile (black) is considered cell cycle dependent. On the bottom panel : nuclear protein shows a nuclear ratio (nuc/total) profile close to 1 most of the cell cycle, while cytoplasmic protein, shows a nuclear ratio close to 0 most of the cell cycle (besides during the mitosis, where the nucleus and cytoplasm are hard to segment apart). Protein that change its localization from the cytoplasm to the nucleus in a cell cycle dependent manner, present a nuclear ratio that is variable across the cell cycle.

To study the cell cycle dependence of protein level and localization, we used the LARC library of human clones with tagged proteins [11], [14], [16]. The library is made of clones based on a parental cell line (H1299 human lung cancer). In each clone, a protein is fluorescently tagged with YFP as an internal exon (Fig1A, B). The protein is tagged in its endogenous chromosomal locus, preserving natural promoter and regulatory sequences. Previous studies suggest that most (∼80%) of the tagged proteins preserve their wild-type dynamics, localizations and levels [11], [16] (see also Material S1 and Fig S1, S2). Notably, in contrast to exogenous expression of tagged proteins, the present system does not lead to protein over-expression. The parental clone also expresses proteins tagged with red florescence (mCherry). The red fluorescence is used for image analysis- allowing automated segmentation of the nucleus and cytoplasm in all clones (Fig 1C) [11].

A previous study followed about 1000 clones with different tagged proteins as they responded to an anti-cancer drug using time-lapse movies [11]. About 100 of the movies also included 24 h before drug addition. Here, we re-analyzed these movies, together with movies from a recent study on protein half-lives using the same cells and microscopy system [16] and chose 495 unique proteins with high quality movies for further analysis (movies chosen had 4 fields of view totaling at least 20 cells at each time-point, and where protein localization matches literature).

Using dynamic proteomics, we tracked the protein level and other parameters of individual cells through time (Fig 1D). We also detected the occurrences of cell divisions. Cell division was detected by a sharp twofold decline in the protein level and by detecting a rounding of the cells before the mitosis (Fig 1C), as described [11] (see also Material S1).

We used in-silico synchronization to place the data on a time-axis which indicates the fraction of time elapsed between cell division. A challenge in the present dataset was that the movies usually do not include, for all cells, a complete cell cycle. The 24 h movies typically contain one cell division event per cell – since the average cell cycle duration is 21±4 hours (Fig S2). Cells also move with a mean speed of about 10 µm/h, and thus some of the cells do not remain in the field of view for the entire movie duration. As a result, the 24 h movies capture only a part of the cell cycle for each cell.

To address this challenge, we arranged the data for each cell on a time axis which measures time from the previous cell division, or time to the next cell division. Time was normalized to the average cell cycle duration (21 h; similar conclusions are found using other values ranging between 18–23 h, see Fig S3). In this way, partial traces that cover only some of the cell-cycle can be combined to yield information on the average cell-cycle behavior of each tagged protein (Fig1E). We tested the validity of profiles generated from partial traces measured in 24 hours by comparing to profiles generated from 48 and 60 hours movies and we got very similar results (See Material S1 and Fig S3). We used this approach to study cell-cycle dependence of protein level and localization (Fig 1F), as described next.

### 11% of Proteins Show Cell-cycle Dependent Changes in their Level

**Figure 2:**
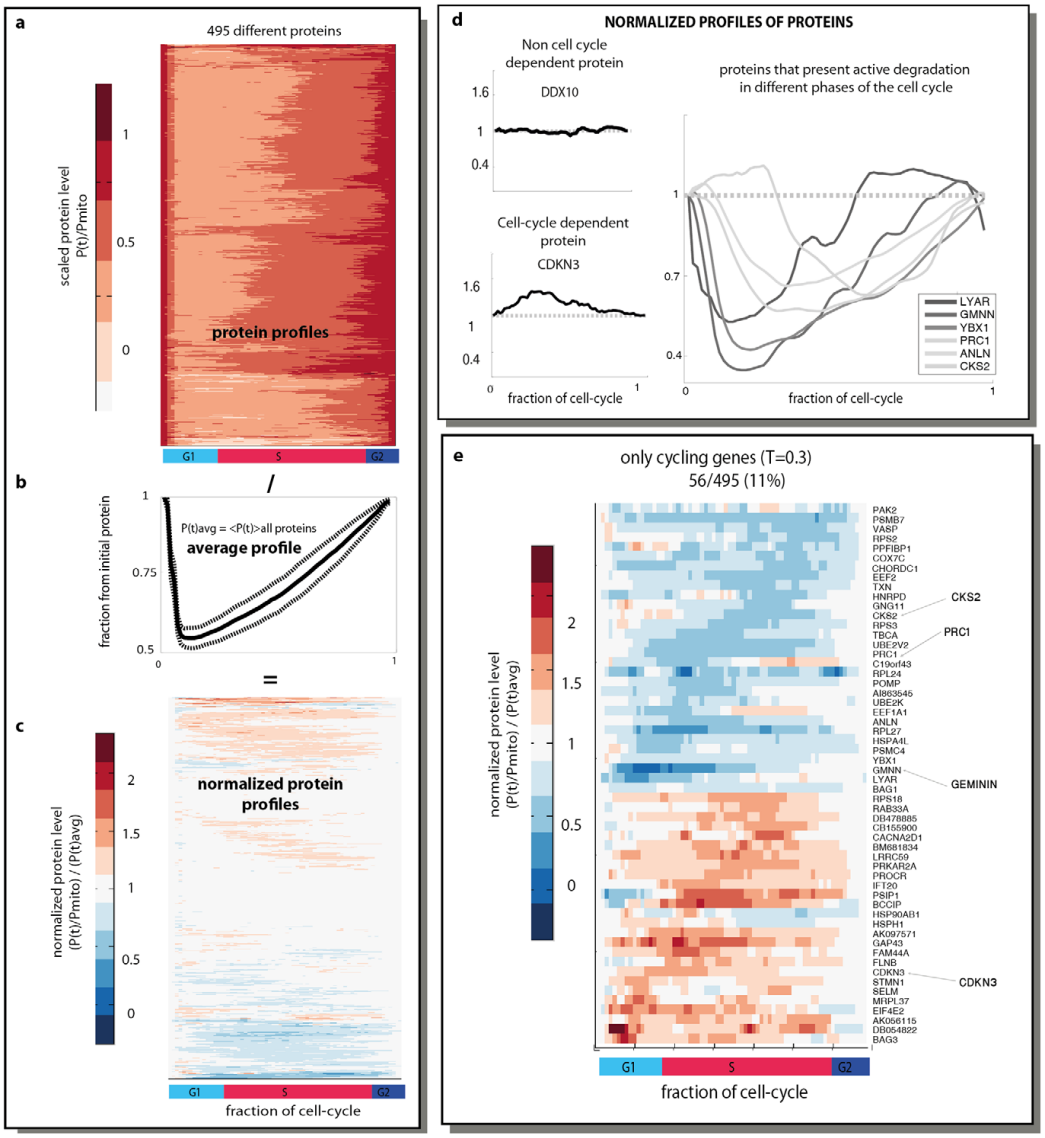
Cell cycle dependence of protein levels. (A) Fluorescence averaged over all cells for 495 proteins, as a function of fraction of cell cycle. Each profile was normalized by the initial level of protein before mitosis to factor out different absolute expression levels. (B) Average profile of over all proteins. Dashed lines represent the standard deviation. (C) Fluorescence profiles normalized by the average profile were clustered using hierarchical clustering. (D) Examples for different clusters of proteins are shown. (E) Normalized profiles of the 56 cycling proteins are presented.

To study cell-cycle dependence of tagged protein levels, we first computed for each protein the fluorescence profile averaged over all cells as a function of the fraction of the cell-cycle, as provided by in-silico synchronization. This is denoted P(τ), where τ is the fraction of the cell cycle, and goes from 0 to 1 indicating the span between two mitosis events. We divided each profile by the fluorescent value at mitosis to factor out differences in absolute fluorescence (Fig 2A).

To determine whether a given protein is cell-cycle dependent, we compared it to the average profile of all proteins, The average profile has a sharp decrease to one half of the initial level upon cell division, followed by gradual, slightly accelerating accumulation until the next division (Fig 2B). This average profile represents the drop of cell volume to one half after division, followed by growth and production of new protein [15], [17]. The average profile serves as a baseline for comparing proteins, since cell cycle dependence is defined as significant deviation from this average behavior – see for example [2], [15].We therefore divided each protein profile by the average profile (Fig 2C). This normalization also factors out systematic errors in the assay, such as effects of cell rounding on measurements of fluorescence.

We next sought to determine criteria for significant cell-cycle dependence. A non-cell-cycle protein follows the average curve, and thus its normalized profile is equal to one at all times. We computed for each normalized protein a score that indicates its deviation from 1. We used the 90% percentile of deviations over time, a score that is tolerant of outlier data (similar results were found also for other scores such as rms distance). To judge the significance of the score compared to experimental noise, we used a bootstrapping approach. We computed the distribution of experimental noise by using data of four proteins with 48 repeat movies each, including day to day repeats [11]– as opposed to only 4 movies for most of the proteins. We used bootstrapping to choose random sets of 4 movies from these 48, and computed the distribution of deviations between repeat normalized profiles from the average profile over all 48 movies. We find that a threshold score of s>0.3 excludes experimental noise at a 99% confidence level (Fig S4).

We find that 11% of the tested proteins (56/495) show significant cell-cycle dependence in their protein level according to our criterion (Fig 2E). These proteins show a wide range of cell-cycle phases, as evaluated by the broad distribution of the position of their peak or trough expression over the cell cycle (Fig S5). GO (Gene Ontology) analysis of the 56 cycling proteins (using the 495 examined proteins as a background) showed that enrichment in the following GO categories: cell cycle regulation (p-value  = 10^−2^), and regulation of kinase activity (p-value  = 10^−2^).

Examples of cell-cycle dependent protein levels are shown in Fig 2E. Known cell-cycle dependent proteins include PRC1 (protein regulator of cytokinesis 1 isoform 1) and GMNN (Geminin), which were found in our analysis to have a sharp drop in level at the beginning of the cell cycle, suggesting active degradation of these protein after mitosis. Indeed, Geminin is known to be a substrate for the Anaphase Promoting Complex (APC), and is degraded by it after mitosis [18]. Several other proteins show similar profiles that suggest active degradation at different phase of the cell cycle (Fig 2E). Geminin and PRC1 also show a peak of expression at G1/S (GMNN) or M/G1 (PRC1) [4]. The cell cycle protein CDKN3 (cyclin-dependent kinase inhibitor 3) shows a peak at G1 phase. Previous studies suggest it shows a peak of mRNA concentration at M/G1- so that a rise in mRNA may precede the rise in protein. Another kinase, CKS2 (CDC28 protein kinase regulatory subunit 2), shows a decrease in protein level in the S phase, suggesting again an active degradation. This gene was found to show cell-cycle dependent mRNA changes, with an increase in mRNA level at G2/M, which is later than the peak observed in protein levels. In general, we do not expect mRNA and protein levels to necessarily correlate, due to translation and degradation modes of control [19]. In summary, several known cell-cycle dependent proteins are captured by the present assay.

We also compared our results to data on global cell-cycle dependent changes in mRNA expression previously measured using different synchronization techniques and microarray measurements [4], [5], [7]. Only a small overlap in mRNA cycling genes was found between Cho and Whitfield studies (about 12% of the cycling genes were common to both studies). Comparison of the present cell cycle dependent proteins list to Whitfield et al. shows a small but significant overlap (7 of the 29 cycling proteins that were measured here and were also investigated in Whitfield et al were evaluated as cycling on the mRNA level (hypergeometric p-value  = 0.02).

### 19% of Proteins Show Cell-cycle Dependent Changes in Nuclear Localization

**Figure 3:**
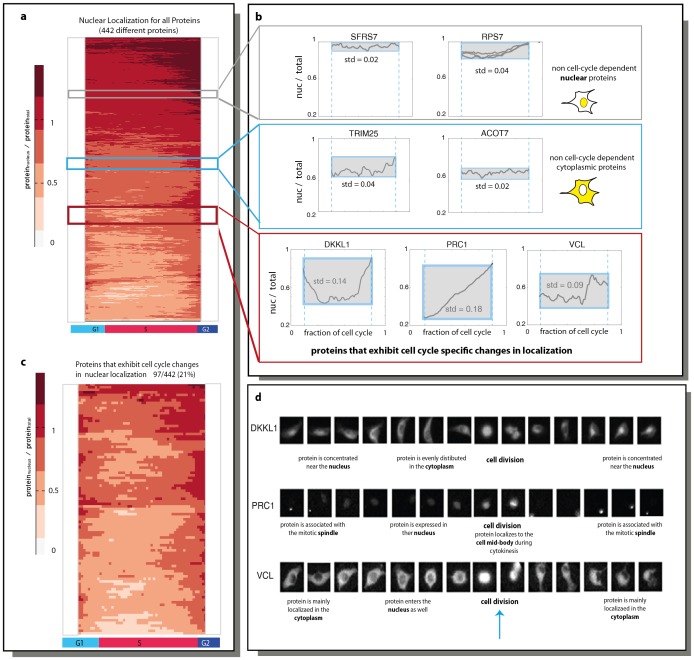
Cell cycle dependence of nuclear localization. (A) The ratio between the fluorescence in the nucleus versus the total fluorescence is shown for 442 proteins as a function of fraction of cell cycle. Due to difficulties with discrimination between the nucleus and the cytoplasm near mitosis, we excluded the values in the first and last 2 hours of the cell cycle. (B) Examples for nuclear, cytoplasmic proteins and proteins that changes localization in a cell cycle specific manner are shown. (B) Profiles of the nuclear ratio of the 97 cycling proteins are shown. (D) Examples of single cells along the cell cycle are shown for three clones that were found to change their localization during the cell cycle. Blue arrow indicates time of cell division.

In addition to protein levels, the present assay provides a means to examine cell-cycle dependence of protein localization. We begin with nuclear ratio (NR), defined by the summed fluorescence over pixels in the nucleus of the cell divided by the summed fluorescence in the entire cell (Fig 3A). Here, it does not make sense to compare a given protein dynamics to the average profile over all proteins, because proteins localized to the nucleus show a very different profile from cytoplasmic ones. Nuclear proteins show a high constant NR, and proteins in the cytoplasm show a low constant NR. In other words, cell-cycle dependence entails a variation over time in NR around the specific mean value for each protein. We therefore used a score which is simply the standard deviation (std) of NR (see examples in the clusters presented in Fig 3A). When computing the std, we removed data from the first and last 10% of the cell cycle, because it is hard to differentiate between nucleus and cytoplasm in these phases due to cell rounding (the nucleus dissolves during mitosis).

We next sought a threshold for the std score that would exclude experimental noise. As above, we used bootstrapping to find a threshold of s>0.08, to exclude experimental noise with 99% confidence (Fig S6).

We find that 19% (96/495) of the tested proteins show cell cycle dependence in nuclear localization (Fig 3B). For example, VCL (Vinculin), a cytoskeletal protein, exhibit entry to the nucleus in G2/M phase (Figures 3C,3D). In addition to VCL`s role in cell-matrix adhesion, it was found to also be a regulator of apoptosis, [20], [21]. VCL was not reported before to localize to the nucleus during the cell cycle, but this localization may relate to the latter functions. Other proteins show more elaborate localization changes such as PRC1, protein regulator of cytokinesis [22], [23]. PRC1 associates with the mitotic spindle during M/G1 and then localizes to the nucleus during the S phase (Figures 3C,3D).

In this dataset, no significant GO enrichment was found. No significant overlap with genes with cell-cycle dependent mRNA was found, which is plausible given that mRNA levels are not generally indicative of localization.

### 23% of Proteins Showed Cell-cycled Dependent Localization Changes, as Measured by Changes in Texture

Finally, we consider changes in protein localization beyond nuclear/cytoplasm ratios. For this purpose, we used an image-analysis approach in which the texture of the cell image is evaluated [24]–[26]. Texture, according to the approach of Haralick [27], is composed of a vector of features that describe the image, such as contrast, granularity and homogeneity.

To calculate texture, we first evaluated a gray-level (fluorescence intensity) co-occurrence matrix (GLCM) from each fluorescent image of the cells [28]. Each element (i,j) in GCLM specifies the number of times that the pixel with gray-level i occurred horizontally adjacent to a pixel with gray-level j.

**Figure 4:**
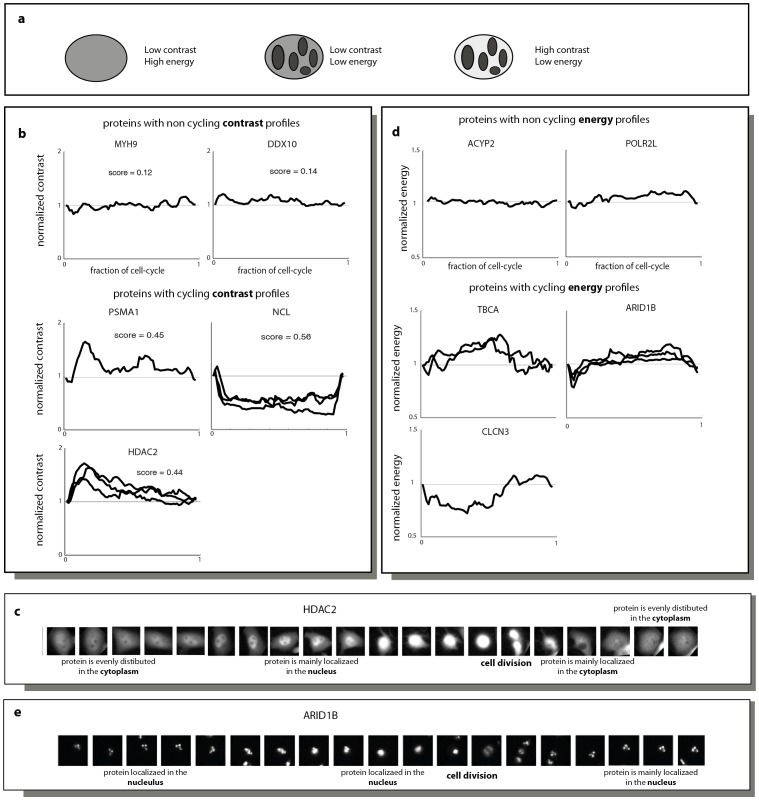
Cell cycle dependence of protein localization using texture features. (A) Illustrations of different cells texture. Note that while the energy ), doesn’t take into account the absolute differences in intensity, those are taken into account in the contrast calculation (, and so, similar cells can have same energy, but different contrast (the 2 cells on the left). (B) Normalized profiles of the contrast along the cell cycle are shown for a few proteins. (C) A series of images from a single cell from a clone that expresses HDAC2 fused to YFP. (D) Normalized profiles of the energy along the cell cycle are shown for a few proteins. (E) A series of images from a single cell from a clone that expresses ARID1B fused to YFP.

From the matrix one can compute the various texture features. For example ‘contrast’ is giving a value of 0 for a constant intensity image and high values when adjacent pixels have different intensity A distinct feature, called ‘energy’, calculates the sum of squared elements in the GLCM (), energy is 1 for a constant image and ranges from 0 to 1. Though contrast and energy are mostly anti-correlated, different images can have similar energy values and different contrast, and vice-versa (Fig 4A). We measured four commonly used features, contrast, energy, homogeneity and correlation (Fig S7), and tracked them over the cell cycle for each protein.

Here, again, we normalized the texture profile of each protein by the average profile and scored deviation from the mean profile using the percentile score described above (see examples in Fig 4B–E). We found that 6% (29/495) of the proteins showed a cell-cycle dependent changes in contrast (threshold  = 0.4), and 15% (76/495) showed a cell-cycle dependent changes in energy (threshold = 0.15). Overall, we find that 23% of the proteins (113/495) show a cell cycle dependent texture profile. A cell cycle dependent change in the texture profile of a protein implies that the protein localizes differently inside the cell in the different phases of the cell cycle. We couldn’t detect a significant category enriched in GO for this set of genes. There was also no enrichment for these genes in the list of cell-cycle dependent mRNAs.

An interesting example is the HDAC2 protein, a histone deacetylase. Histone acetylation is known to play an important role in the regulation of gene expression. This protein is evenly distributed in the cytoplasm during G1, and then enters the nucleus during S/G2 phase. Right after mitosis, the protein is again excluded from the nucleus. These changes in protein localizations would be interesting to explore, since it can indicate different roles for this protein in the cell cycle. It has been reported before, that HDAC class 2 family of proteins shuttle between the nucleus and cytosol, and their subcellular localization is affected by protein-protein interactions [29], [30].

### In Total, 40% of the Proteins Show Cell Cycle Dependent Localization and/or Levels

**Figure 5:**
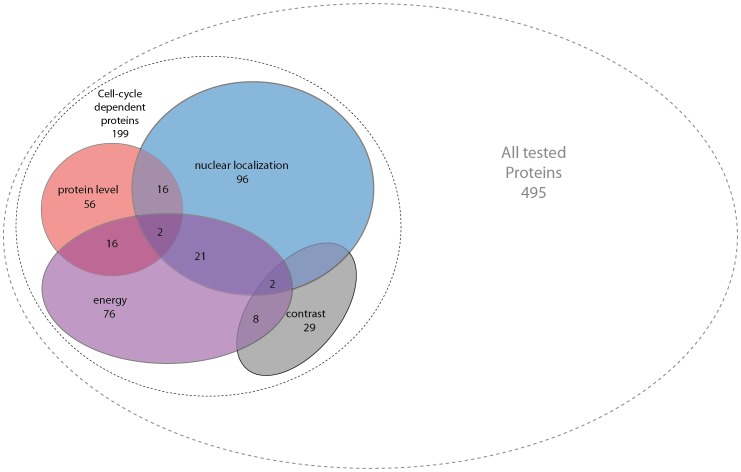
Distribution of cell cycle dependent proteins with changes in level and localization. The number of proteins that were found to be cell cycle dependent in each of the tested categories is shown and so are the number of proteins that were found to be cell cycle dependent in several categories.

Out of the 495 proteins in this study, 40% of proteins (40%, 199/495) show cell cycle dependence in levels or localization (Fig 5). Only about 7% (34/495) show cell-cycle dependence in both levels and localization. Table S1 includes the full dataset of protein profiles and scores.

### Cell-cycle Dependent Expression of Proteins doesn’t Correlate with Replication Timing of the Gene

Each gene or locus in the genome replicates its DNA in a typical time during S phase [31]. Housekeeping genes usually replicate early, whereas low expressed genes or tissue specific genes tend to replicate late [32]. Recent genomic studies mapped the replication timing of all the genome in different cell types[33]–[35]. We tested whether the time that the DNA for a given gene replicates during the cell cycle correlates with the dynamics of its accumulation. We detected no significant correlation between DNA replication timing and changes in accumulation of the corresponding proteins (Fig S8). A recent study (Yunger et al. 2010) that employed high-resolution transcription measurements showed a drastic reduction in promoter firing after replication compared to the rate before replication. This observation may explain why doubling the gene copy number does not lead to a visible increase in protein level.

## Discussion

This study used dynamic proteomics to study the cell-cycle dependence of protein levels and localization of 495 proteins, which represent most classes of proteins in the proteome. Our use of in-silico synchronization rather than perturbative methods for synchronization, and the high accuracy of the single cell measurements, allowed a sensitive assay of cell-cycle dependence of proteins expression and localization.

We find that 40% of the proteins showed cell cycle dependence in level and/or localization. Most of the cell-cycle dependence was in localization (89% of the cell-cycle dependent proteins). Of these, 48% in nuclear/cytoplasmic ratios and 57% in texture (16% of the cell-cycle dependent proteins are combined to these 2 categories).

Even if one considers only protein level, not localization, one finds that about 11% of the proteins are cell-cycle dependent; This exceeds by far the estimates based on global mRNA studies which suggested that 1–3% of mRNAs are cell-cycle dependent [4], [5], [7]. Moreover, since we chose a stringent threshold in all our analyses that excludes experimental noise at a 99% confidence level, we believe that the presented percentage of cycling proteins is an underestimation.

Localization change is an efficient mode of regulation: it is rapid since no new protein need be synthesized. Recently, it was shown that changes in the localization of Cdk1-cyclin B1 during the cell-cycle include a positive feedback and ensures a rapid, complete, robust, and irreversible transition from interphase to mitosis [36]. Dynamic proteomics is almost unique amongst proteomic assays in the ability to assay localization changes of endogenously expressed human proteins over time at high accuracy. In our system, the nucleus and cytopslam are labeled by mCherry and can be segmented. Therefore, we can automatically identify proteins that move from the nucleus to the cytoplasm in a cell cycle dependent manner. Other localization changes of proteins are hard to detect automatically in a large-scale study, since other organelles of the cell are not labeled and cannot be segmented. In this study, we used different texture features that describe the cell granularity to allow the automatic detection of such changes.

Of the nuclear proteins in our study, 6.5% showed cell cycle dependence in levels. This is lower than the 40% found in a sample of 20 nuclear proteins by Sigal et al. This difference may stem from a low number of proteins tested in the former study or from a more stringent threshold in our study. The fraction of cytoplasmic proteins with cell-cycle dependent expression was higher in our study than the fraction of cell-cycle dependent nuclear proteins [6.5% (8/124) vs. 17% (71/137) - P<0.0001, Fisher`s exact test].

It would be interesting to expand the set of protein tested to see whether cell cycle dependence is as widespread as suggested by the present sample. Furthermore, since dynamic proteomics allows studying individual cells, it would be interesting to assay the cell-cell variability in cell-cycle profiles. This is unfeasible with the present dataset due to the length of movies. Finally, it would be important to understand the mechanisms that lead to cell-cycle changes in localization and levels, to better understand the changes that a human cell undergoes as it transits through the cell cycle.

## Materials and Methods

### Time-Lapse Microscopy Movies

More than 4,000 movies from previous studies (A. A. Cohen et al. 2008; Eran Eden et al. 2011) were used for this analysis. In the previous studies, 4 movies (fields of view) were taken for each of the 1,000 clones totaling in about 4,000 movies. In each movie, 10–20 cells were tracked over 24 hours at least, every 20 minutes. Some of these clones were filmed more than once and some clones represent the same protein. We averaged the protein profile over 4 fields of view. For the final analysis, we combined all data for the same protein from all relevant movies. We observed a high reproducibility of the protein profiles calculated from day to day repeats for single protein (see Figures 3C, 4B and 4E). Each time point included transmitted light image (phase contrast) and two fluorescent channels (red and yellow).

### Image Analysis of Time-Lapse Movies

We used the image analysis software described in Cohen et al. (2008) with minor modifications. The main steps in this software include background correction (flat field and background subtraction), segmentation, cell tracking, and automated identification of cell phenotypes (mitosis and cell death). Cell and nuclei segmentation was based on the red fluorescent images of the red tagged protein found in all clones, localized to the cytoplasm and nucleus, with intensity which is very uniform across cells and clones. Segmentation used global image threshold and seeded watershed segmentation. The cell-tracking procedure maps each cell to the appropriate cells in the preceding and following frames as described [37]. Automated cell division identification algorithm utilized the morphological changes correlated with dividing cells and the sharp two fold drop in total fluorescent level between consecutive images. Texture parameters (contrast, correlation, homogeneity and energy) of the proteins were measured for each cell in each time point based on the YFP image of the tagged protein.

### In-Silico Synchronization and Profile Normalization

All mitotic events in a movie were automatically identified as described above. We divided the time of each measurement by the duration of the average cell cycle (21 hours) to get a relative time in the cell cycle (Fig S10).

We divided each profile by the fluorescent value at mitosis, to factor out differences in absolute fluorescence. Both these normalizations result in a typical profile that starts from 1 at time point 0, decrease after division to about 0.5 and accumulated during the cell cycle to 1 again at time point 1 (see Fig 2B). A different approach was used in Ball et al. paper [8], where instead of the total protein, the median pixel was used as an indicator for the concentration of protein in a frame. The total protein is not an indicator for the concentration of protein and is indeed influenced by the increase in the cell size, nevertheless, it sums the protein expression in all the cell even if it is expressed non homogenously and is less influenced by local changes or outliers. We repeated our analysis for the cell-cycle dependency of protein level using the median pixel of cell. We found the median pixel to be a noisier measure (Fig S9), and decided on a similar threshold of score = 0.3 for cell-cycle dependent proteins. Although a significant number of proteins that showed cell cycle dependency using the ‘total protein’ got a high score when using the ‘median pixel’ measure, not all known cell-cycle proteins got high score. (see Table S1).

### Analysis of Cell-Cycle Dependence Changes in Protein Level

The average profile over all the protein profiles was calculated by calculating the average normalized value at each time point. The profiles were then normalized by dividing with this average profile. A clone that its profile is very similar to the average profile is not considered a cell-cycle dependent protein and its deviation from the average profile should be small. However, a cell-cycle dependent protein would have a profile that is different from the average profile of all proteins in at least one phase of the cell cycle and so its deviation from the average profile would be large. To estimate the extent of deviation of each protein profile form the average profile, we used 2 measures. The first score, S, was the 90% percentile of the deviation of the normalized profile from a constant vector of one. The second score was the RMS, were we calculated the root mean square deviations from the vector of ones. Cell cycle dependent proteins are expected to give high scores.

In order to find criteria for significant cell-cycle dependence, we used a bootstrapping approach. We computed the distribution of experimental noise by using data on four proteins (DDX5, PRC1, MSN and RPS24) for which 48 repeat movies, including day-day repeats, was available [11], [16]. We used bootstrapping to choose 100 random sets of 4 movies from these 48, and computed the distribution of deviations between the 100 repeat normalized profiles from the average profile over all 48 movies.

### Changes in Nuclear Localization

Both the protein in the nucleus and the total protein are measured in our analysis using the accurate segmentation of the cytoplasm and the nucleus [11]. The ratio between the protein in the nucleus and the total protein was calculated for each protein along the cell cycle similarly to the above.

Here, again, we used the bootstrapping approach, generated 100 random sets for each of the 4 proteins and calculated the ratio of nuc/total. The width of the histograms of the std was in all cases smaller than 0.8, and we chose this to be the threshold for cell-cycle dependent nuclear localization.

### Other Localization Changes

Similar approach for studying the protein level changes was taken for all 4 texture features – contrast, energy, correlation and homogeneity. The average profile was calculated and the score was later determined using the bootstrapping approach. 29 proteins (6%) exhibit cell-cycle dependent changes in contrast (Tcn >0.4) and 76 proteins (15%) exhibit cell-cycle dependent changes in energy (Te >0.15). Very few proteins were found to show cell-cycle dependent changes in homegenity (36/495 7%) and correlation (14/495 2.8%) (Th >0.02 and Tcr >0.05 accordingly).

### Other Data Analysis

GO enrichment analysis was done using the WEBGESTALT [38]. Cell-cycle dependent changes in mRNA were taken from Whitfield et al. [4], hyper-geometric p-values were calculated in matlab.

## Supporting Information

Figure S1 **Cell and nucleus size distribution.** Results for 11 more clones are summarized in the table. The cell size ranges from 1100 to 1600, where the std of the cell size for each clone is about 500. The nucleus size is between 400–600, where the std is about 170. Differences between different clones seem to be in the range of the standard deviation.
(TIF)

Figure S2 **Distribution of durations of the cell cycle of several clones**. The cell cycle duration varies from 19 to 24 hours in all the examined clones.
(TIF)

Figure S3 **Cell-cycle dependent profiles of PRC1 and DDX5 using different parameters**. Profiles of DDX5 and PRC1 along the cell cycle are shown. The Tt stands for the cell cycle duration that was used for cells where only partial tracks exist and is used for normalization of the relative time in the cell cycle. different values of Tt were tested (top panel - 18 hours, middle panel - 21 hours, bottom panel - 23 hours) and exhibit very similar profiles. In each panel and for each clone, only a fraction of the 60 hours movie was used (18 h–60 h) to estimate differences in profile that stems from short time frame of observations. Note that profiles that are generated from 24 hours movie are highly identical to profiles generated from 48 and 60 hours movie.
(TIF)

Figure S4 **Bootstrapping approach was used to determine the threshold for cycling genes**. Data from four different proteins, each with 48 repeat movies was used to generate histograms of experimental deviation between profiles. For each protein, 4 different movies were chosen randomly from the set of 48 and a profile was generated. A score was calculated (90th percentile of the deviation from the average vector of the 48 movies) between each profile and the average profile and a histogram of scores was generated. Given these histograms, a threshold distance of 0.3 was determined to exclude 99% of the experimental variation.
(TIF)

Figure S5 **Histograms of the position of the maximum or minimum expression of the cell cycle dependent proteins are depicted.**
(TIF)

Figure S6 **Bootstrapping approach was used to determine the threshold for cycling nuclear localizations.** (A) We used similar approach to the described in Figure S1. The ratio Nuc/total is plotted along the cell cycle for 4 proteins. For each protein, a profile was calculated based on 4 movies that were randomly picked from a group of 48 movies, 100 times. (B) The std of the nuc/total ratio along the time was calculated (after we removed data from the first and last 10% of the cell cycle) for each protein and the histograms of the 100 simulations is shown. PRC1 is known to change localization during cell cycle and has std values ranging from 0.15 to 0.22. We chose as a threshold, std of 0.08 (Tstd), since DDX5, RPS24 and GMNN shows std values lower than 0.08 in 99% of cases.
(TIF)

Figure S7 **Properties of gray-level co-occurrence matrix.** (Haralick texture features), see: Haralick R, Dinstein & Shanmugan K (1973) Textural features for image classification. IEEE Transactions on Systems, Man, and Cybernetics SMC-3∶610–621.
(TIF)

Figure S8 **Cell-cycle dependent expression of proteins doesn’t correlate with replication timing of the gene.** Genes were sorted based on their time of replication during the S phase (taken from : Farkash-Amar S, Lipson D, Polten A, Goren A, Helmstetter C, Yakhini Z & Simon I (2008) Global organization of replication time zones of the mouse genome. Genome Res. 18∶1562–1570). Note that there is no evident pattern indicating that early gens accumulates protein earlier than late genes.
(TIF)

Figure S9 **Analysis of cell-cycle dependency of protein level using the median pixel.** On the left, the median average profile is shown when using the median pixel. On the right, Bootstrapping approach was used to determine the threshold for cycling genes based on median pixel. Similar analysis to the analysis descibed in Figure S1 was done here for protein profiles based on the median pixel instead of the total protein. The score of the 90th deviation from the mean profile was calculated for the 100 sets of 4 FOVs (Fields of View). Given these histograms, a threshold distance of 0.3 was determined to exclude 95% of the experimental variation.
(TIF)

Figure 10 **Normalization of partial tracks of single cells.** This example illustrates how partial tracks of a single cell that divided 8 hours after the beginning of the movie to 2 daughter cells were used. The partial tracks were normalized to the average cell cycle (21 hours) and overlaid on the cell-cycle profile accordingly.
(TIF)

Table S1 The Supplementary table S1 includes the full dataset of protein profiles and scores.
(XLSX)

Material S1 The online Supplementary material includes additional information about the methods and results presented above.
(DOCX)
