# Supplementary figures and images for: Dynamic Proteomics of Human Protein Level and Localization across the Cell Cycle
Source: PLoS One. 2012 Nov 7;7(11):e48722. doi: 10.1371/journal.pone.0048722 (PMC3492413; doi:10.1371/journal.pone.0048722)

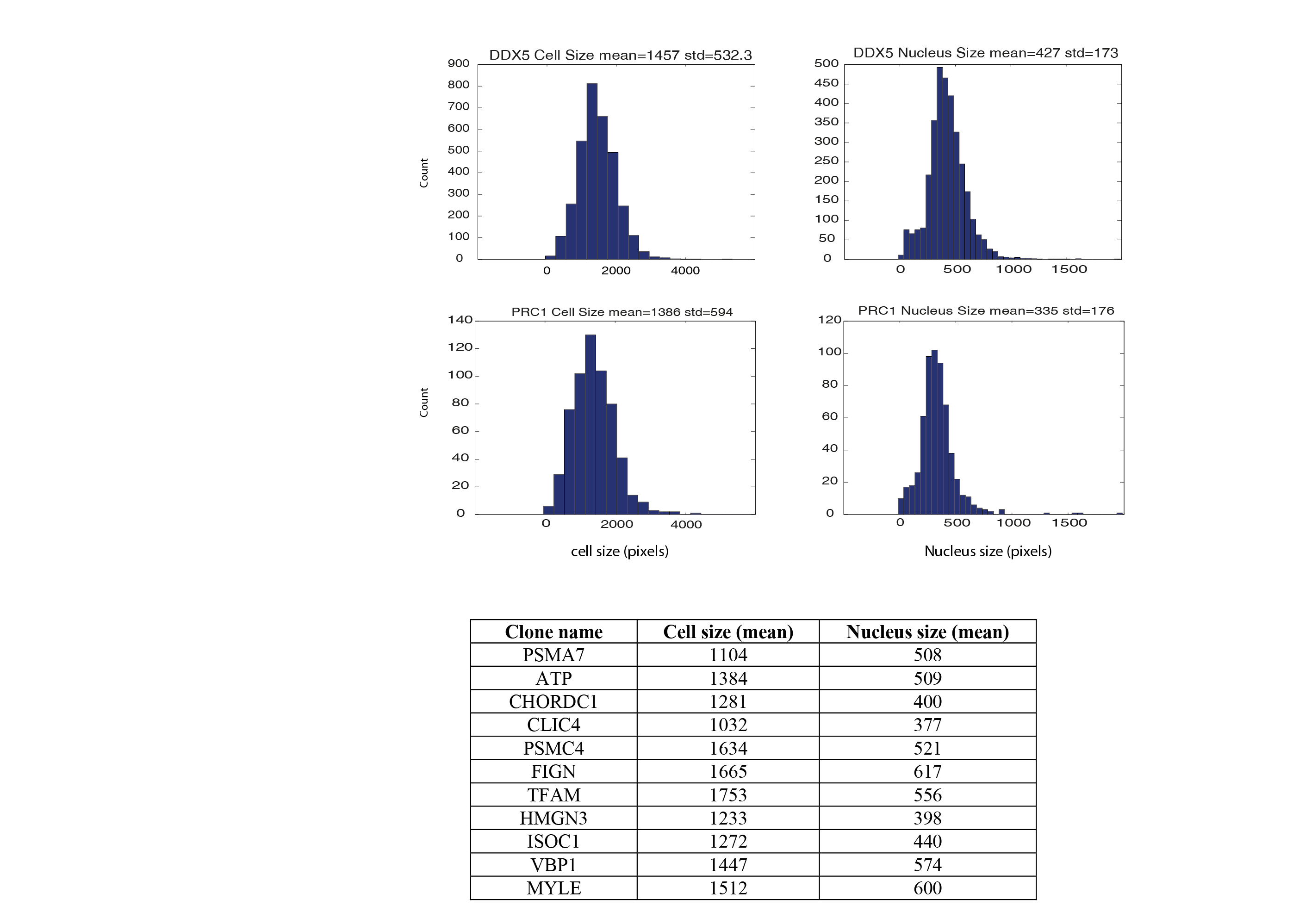

Supplement: Figure S1 — Cell and nucleus size distribution. Results for 11 more clones are summarized in the table. The cell size ranges from 1100 to 1600, where the std of the cell size for each clone is about 500. The nucleus size is between 400–600, where the std is about 170. Differences between different clones seem to be in the range of the standard deviation. (TIF) [file pone.0048722.s001.tif]

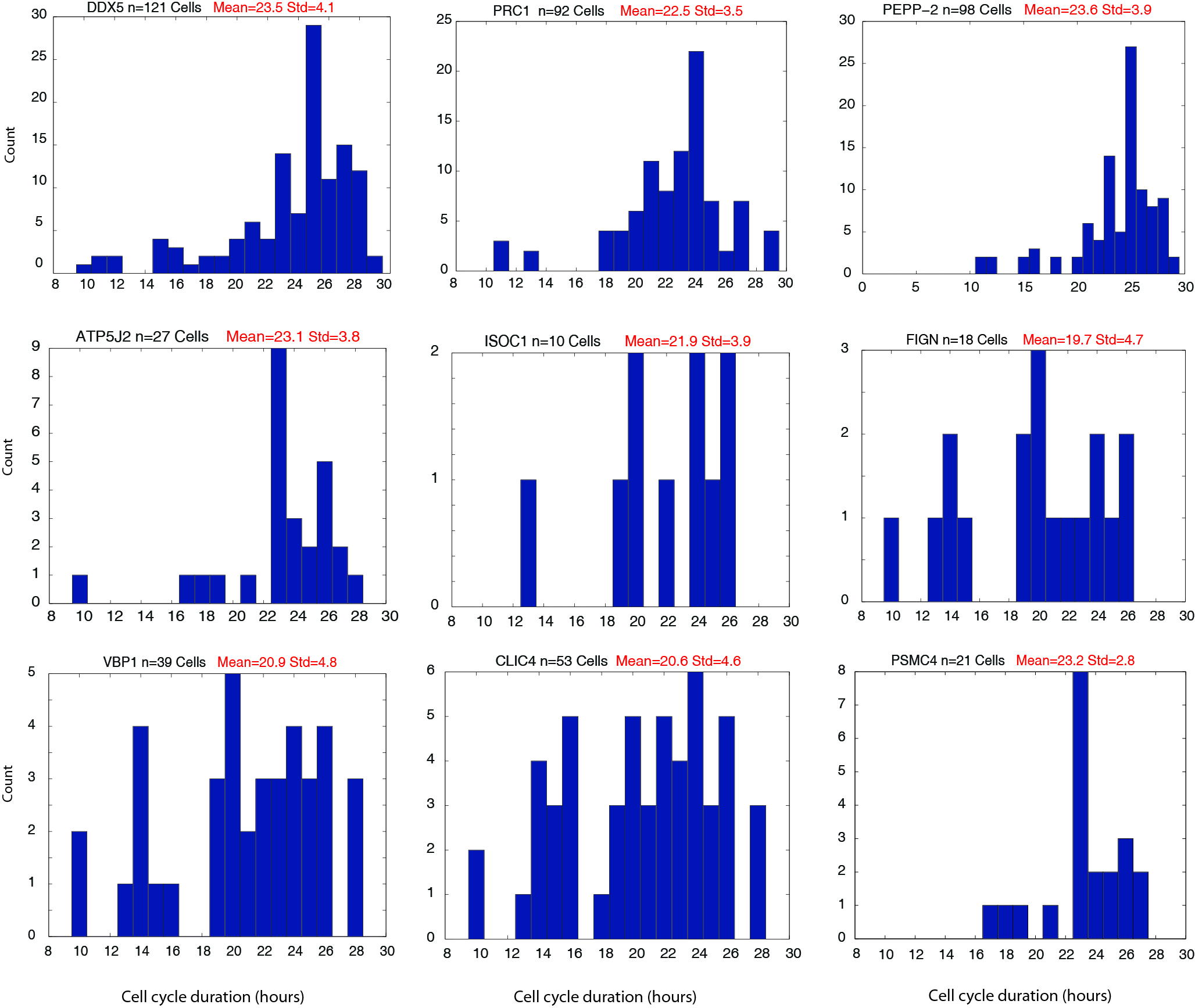

Supplement: Figure S2 — Distribution of durations of the cell cycle of several clones. The cell cycle duration varies from 19 to 24 hours in all the examined clones. (TIF) [file pone.0048722.s002.tif]

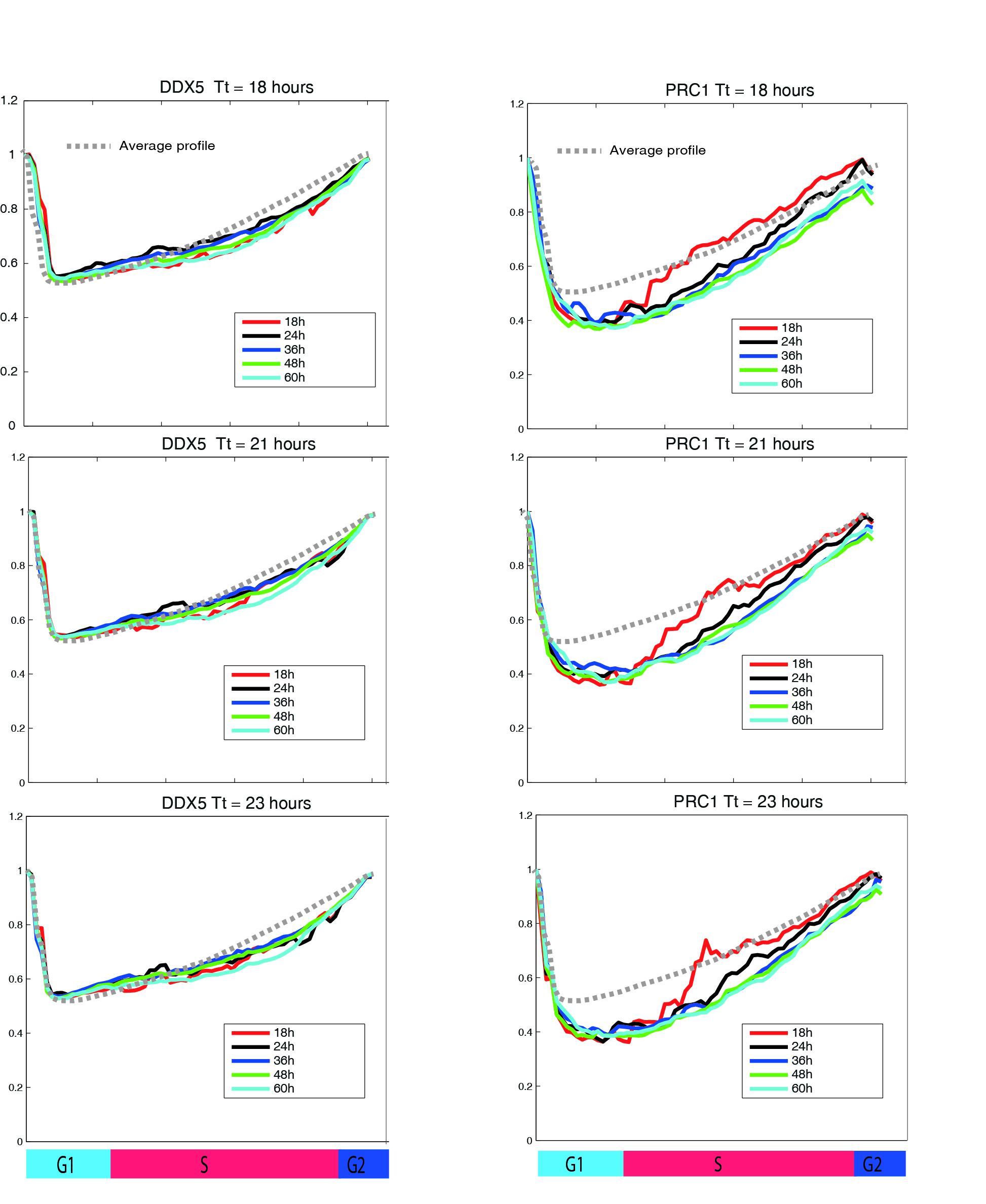

Supplement: Figure S3 — Cell-cycle dependent profiles of PRC1 and DDX5 using different parameters. Profiles of DDX5 and PRC1 along the cell cycle are shown. The Tt stands for the cell cycle duration that was used for cells where only partial tracks exist and is used for normalization of the relative time in the cell cycle. different values of Tt were tested (top panel - 18 hours, middle panel - 21 hours, bottom panel - 23 hours) and exhibit very similar profiles. In each panel and for each clone, only a fraction of the 60 hours movie was used (18 h–60 h) to estimate differences in profile that stems from short time frame of observations. Note that profiles that are generated from 24 hours movie are highly identical to profiles generated from 48 and 60 hours movie. (TIF) [file pone.0048722.s003.tif]

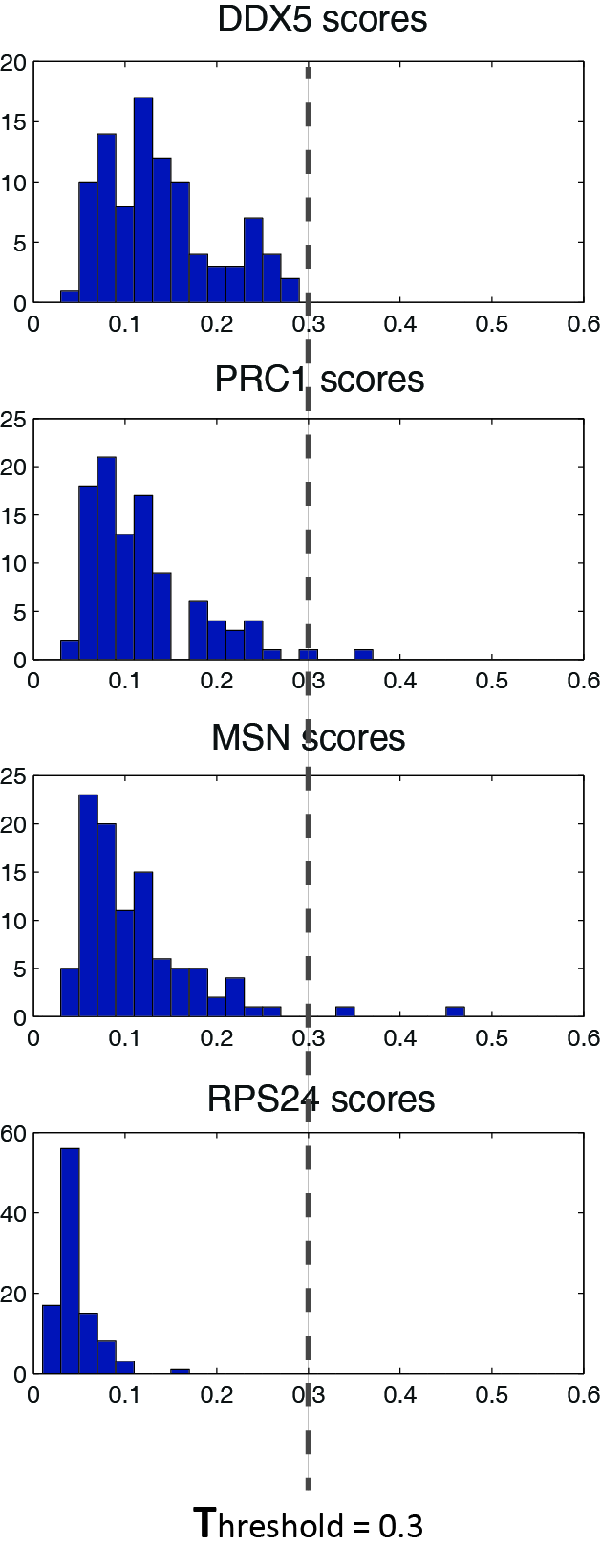

Supplement: Figure S4 — Bootstrapping approach was used to determine the threshold for cycling genes. Data from four different proteins, each with 48 repeat movies was used to generate histograms of experimental deviation between profiles. For each protein, 4 different movies were chosen randomly from the set of 48 and a profile was generated. A score was calculated (90th percentile of the deviation from the average vector of the 48 movies) between each profile and the average profile and a histogram of scores was generated. Given these histograms, a threshold distance of 0.3 was determined to exclude 99% of the experimental variation. (TIF) [file pone.0048722.s004.tif]

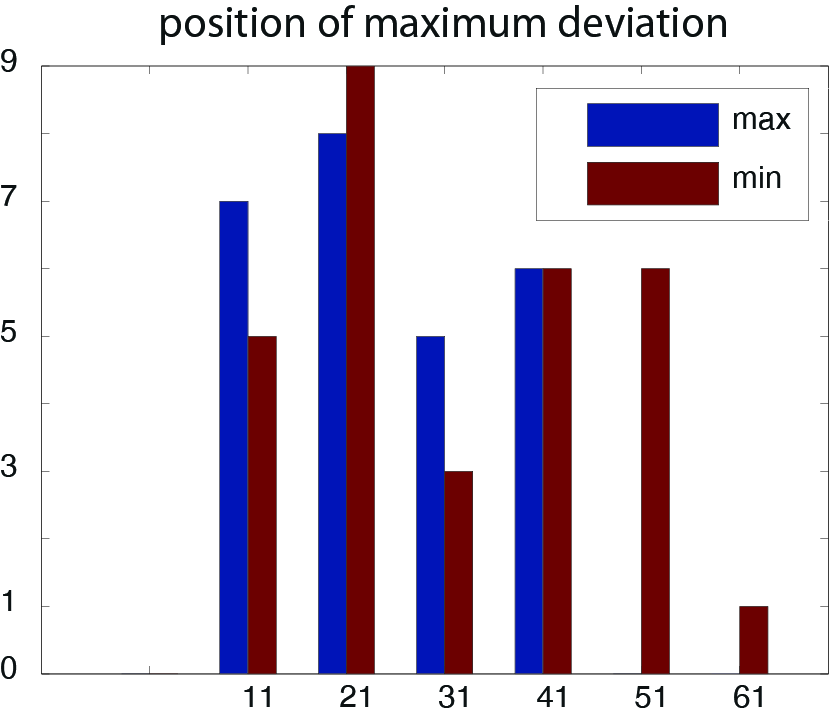

Supplement: Figure S5 — Histograms of the position of the maximum or minimum expression of the cell cycle dependent proteins are depicted. (TIF) [file pone.0048722.s005.tif]

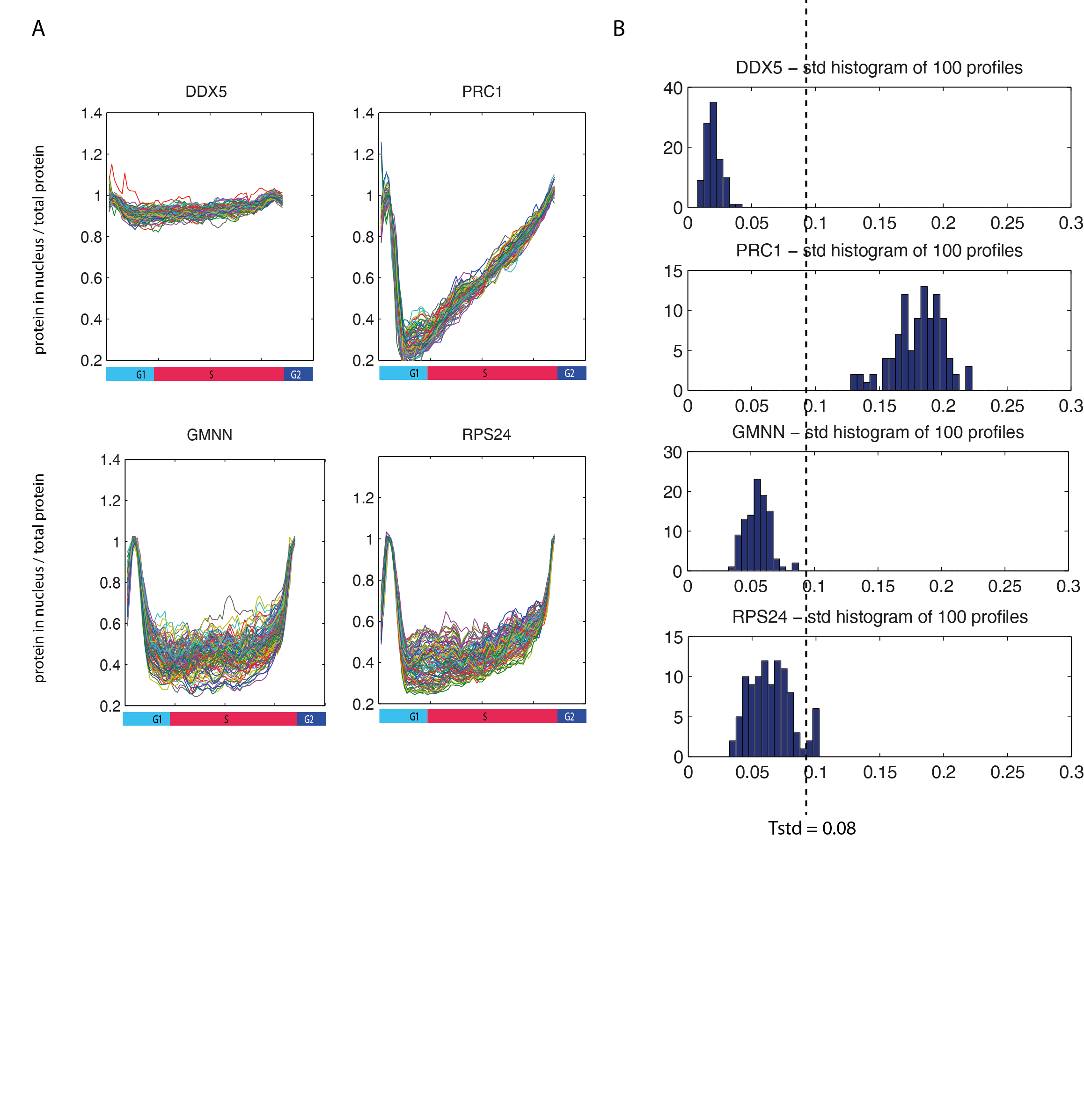

Supplement: Figure S6 — Bootstrapping approach was used to determine the threshold for cycling nuclear localizations. (A) We used similar approach to the described in Figure S1. The ratio Nuc/total is plotted along the cell cycle for 4 proteins. For each protein, a profile was calculated based on 4 movies that were randomly picked from a group of 48 movies, 100 times. (B) The std of the nuc/total ratio along the time was calculated (after we removed data from the first and last 10% of the cell cycle) for each protein and the histograms of the 100 simulations is shown. PRC1 is known to change localization during cell cycle and has std values ranging from 0.15 to 0.22. We chose as a threshold, std of 0.08 (Tstd), since DDX5, RPS24 and GMNN shows std values lower than 0.08 in 99% of cases. (TIF) [file pone.0048722.s006.tif]

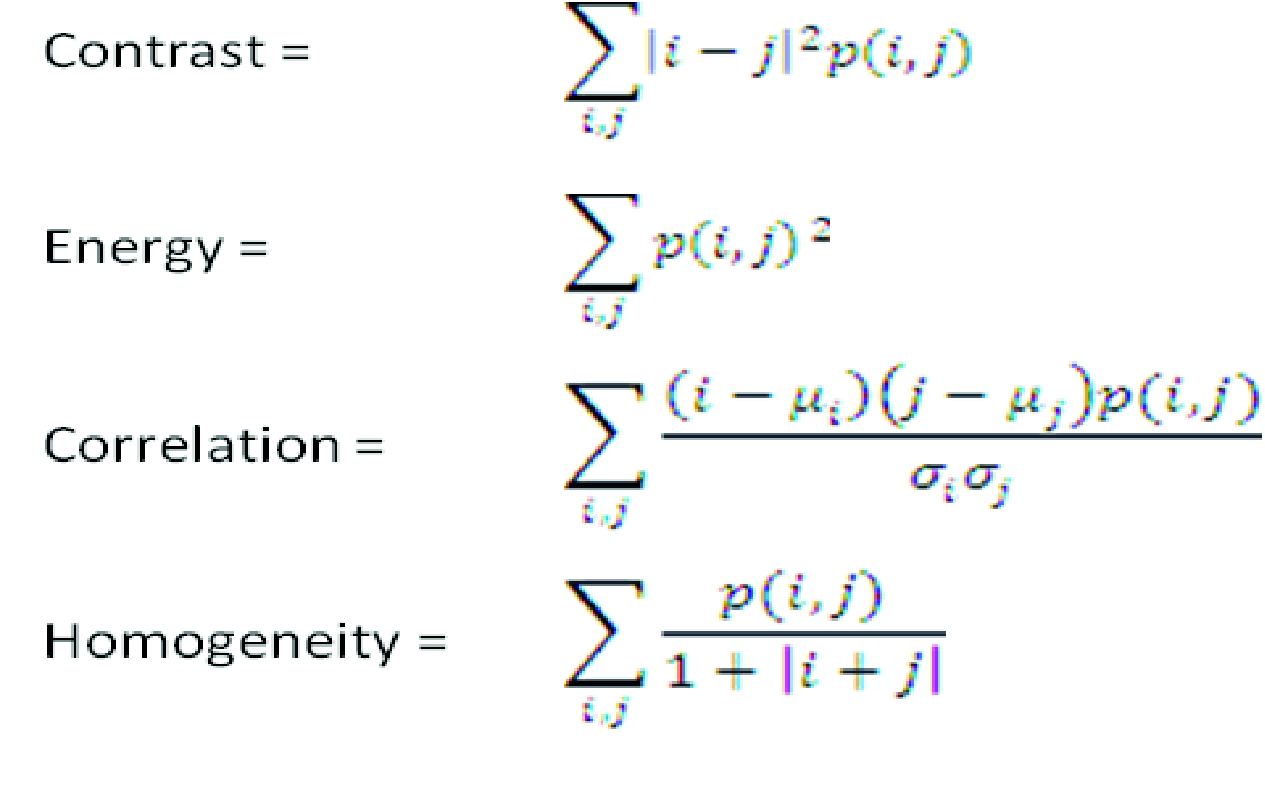

Supplement: Figure S7 — Properties of gray-level co-occurrence matrix. (Haralick texture features), see: Haralick R, Dinstein & Shanmugan K (1973) Textural features for image classification. IEEE Transactions on Systems, Man, and Cybernetics SMC-3∶610–621. (TIF) [file pone.0048722.s007.tif]

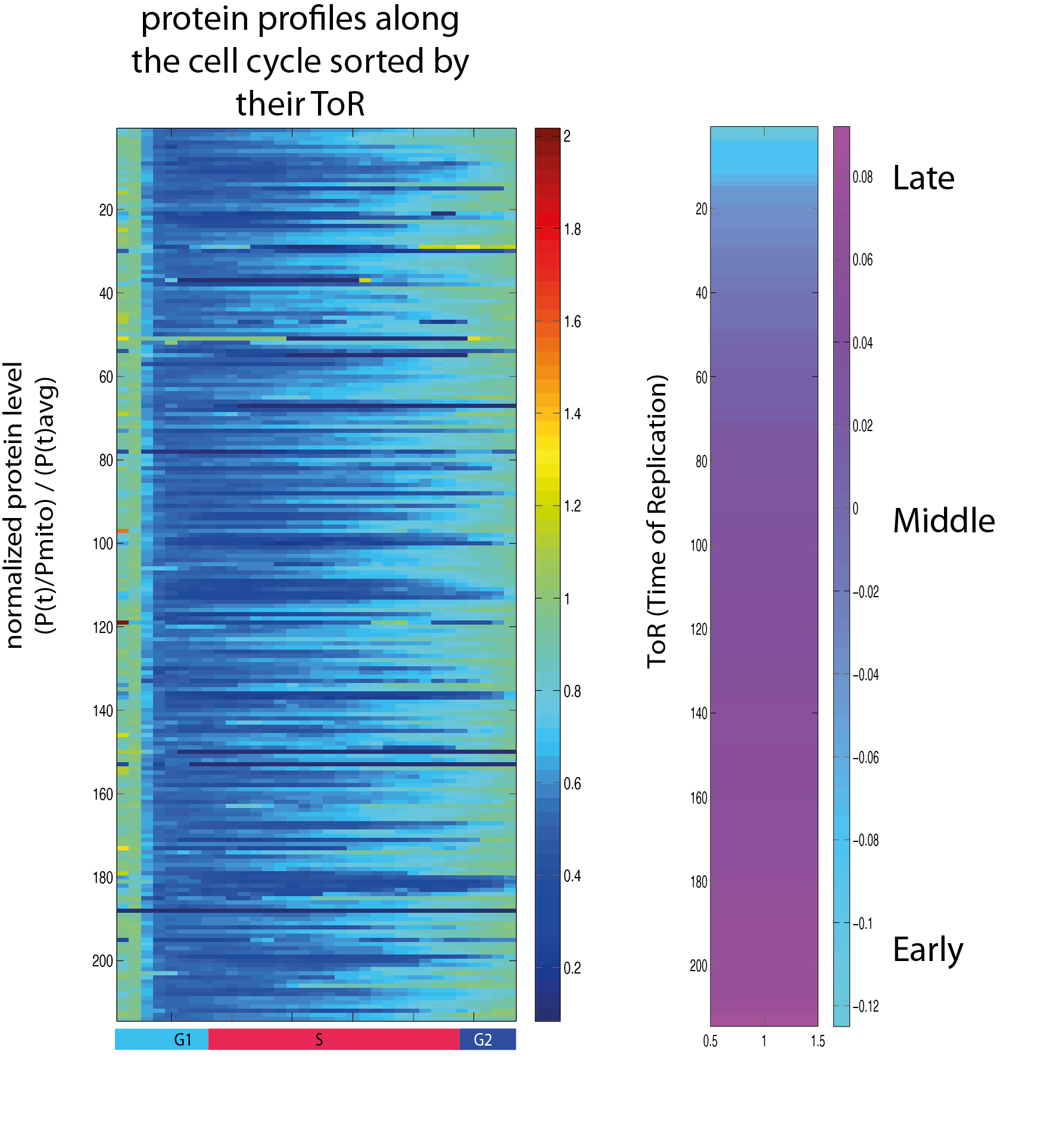

Supplement: Figure S8 — Cell-cycle dependent expression of proteins doesn’t correlate with replication timing of the gene. Genes were sorted based on their time of replication during the S phase (taken from : Farkash-Amar S, Lipson D, Polten A, Goren A, Helmstetter C, Yakhini Z & Simon I (2008) Global organization of replication time zones of the mouse genome. Genome Res. 18∶1562–1570). Note that there is no evident pattern indicating that early gens accumulates protein earlier than late genes. (TIF) [file pone.0048722.s008.tif]

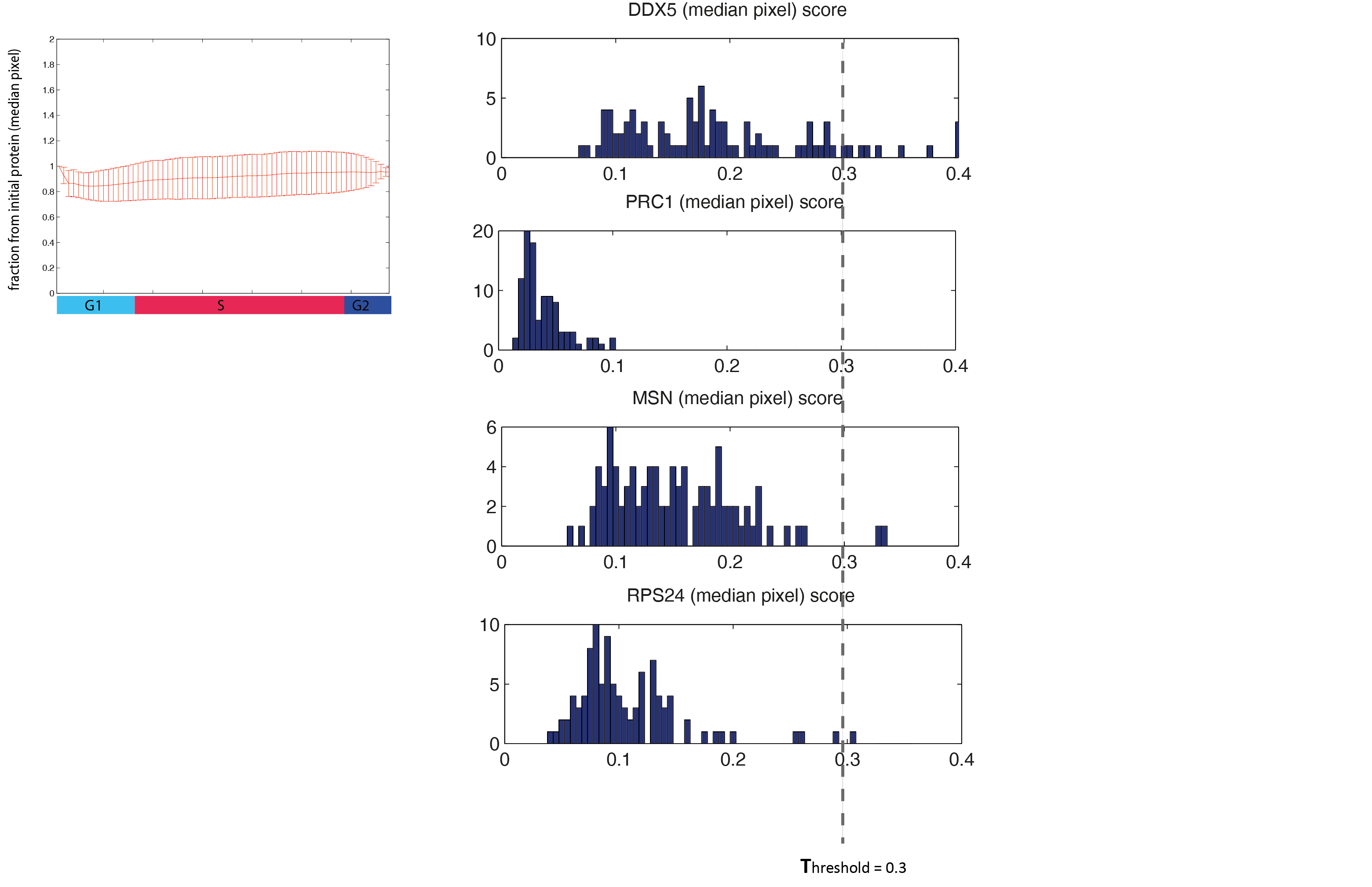

Supplement: Figure S9 — Analysis of cell-cycle dependency of protein level using the median pixel. On the left, the median average profile is shown when using the median pixel. On the right, Bootstrapping approach was used to determine the threshold for cycling genes based on median pixel. Similar analysis to the analysis descibed in Figure S1 was done here for protein profiles based on the median pixel instead of the total protein. The score of the 90th deviation from the mean profile was calculated for the 100 sets of 4 FOVs (Fields of View). Given these histograms, a threshold distance of 0.3 was determined to exclude 95% of the experimental variation. (TIF) [file pone.0048722.s009.tif]

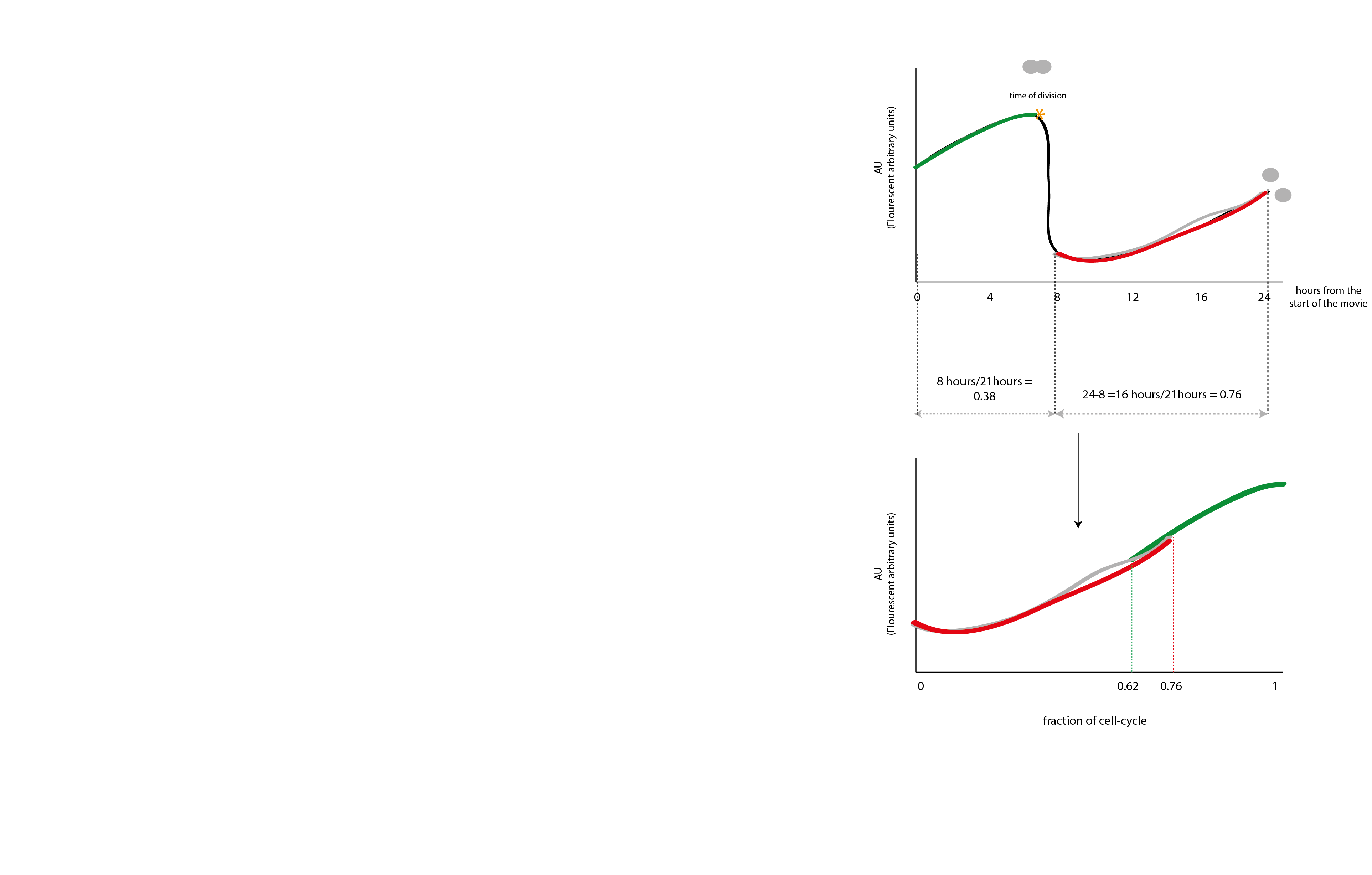

Supplement: Figure 10 — Normalization of partial tracks of single cells. This example illustrates how partial tracks of a single cell that divided 8 hours after the beginning of the movie to 2 daughter cells were used. The partial tracks were normalized to the average cell cycle (21 hours) and overlaid on the cell-cycle profile accordingly. (TIF) [file pone.0048722.s010.tif]
