# Supplementary material for: Dynamic Proteomics of Human Protein Level and Localization across the Cell Cycle
Source: PLoS One. 2012 Nov 7;7(11):e48722. doi: 10.1371/journal.pone.0048722 (PMC3492413; doi:10.1371/journal.pone.0048722)
Supplement: Material S1 — The online Supplementary material includes additional information about the methods and results presented above. (DOCX) [file pone.0048722.s012.docx]

**Online Supplementary material to Dynamic proteomics of human protein level and localization across the cell cycle**

Shlomit Farkash-Amar, Eran Eden, Ariel Cohen, Naama Geva-Zatorsky, Lydia Cohen, Ron Milo, Alex Sigal, Tamar Danon, Uri Alon

Gene Tagging

Tagging of proteins can cause deleterious effects. In order to avoid that, we only used clones that presented similar protein localization to their known localization. Furthermore, in the paper of Eden et al. [1] it was shown that protein half‐lives of YFP‐tagged and untagged proteins are similar. In another paper from our lab, Cohen et al. [2] (Science 2008) showed by Immunoblots that twenty examined proteins show dynamics highly similar to those in the library movies in response to the drug CPT, again suggesting similar functionally to the tagged proteins in our library. In this study, we further compared the division times of different clones that were tracked for 48 hours (Fig S1) and also the distribution of the cell size and the nucleus size for several clones (Fig S2). Since the cell size and the division times between clones were similar, we believe that our tagging of the proteins doesn’t influence dramatically their cell cycle dependency.

Time of Cell Division

Time of division is predicted based on the rounding of the cell, a feature that is seen clearly in the phase images (as demonstrated in Fig 1C) and is automatically detected using a learning algorithm (ANN - Artificial neural network) . We also demanded a decrease in total protein of about two fold. If a protein has a low expression during mitosis, we should still be able to see 2 fold decreases after division to 2 daughter cells.

Short time frame of the observations

In the paper, we used data that was collected for single cells during 24 hours. For most cells, we couldn’t detect 2 consecutive cell divisions since the division time is about 21-23 hours and cell move in and out of the frame. We therefore collected also partial tracks and used the average cell cycle duration (21 hours) to normalize the time of observations to relative time in the cell cycle. We realize that it may introduce a bias, since there is variability in the cell cycle duration between different cells (See Fig S2). However, when calculating the cell-cycle profile for 2 clones with different cell-cycle duration average time of 18h, 21h and 23 hours, we got very similar profiles (Fig S3) and so we believe that this bias doesn’t change much the results.

Furthermore, we compared profiles generated from 24 hours movie to profiles generated from 48 and 60 hours movies for the 2 examined clones (Fig S3). We got very similar profiles and so we believe that using this partial data and the normalization we performed was useful in the case that many full cell cycle tracks (single cells where 2 consecutive mitosis events are apparent) are hard to measure.

Data Analysis

Recently, time-lapse fluorescence microscopy was used to study the cell-cycle dynamics of 16 yeast proteins [33]. Ball et al. used spectral subtraction to remove noise and then calculated the amplitude and period of the oscillation based on the time between local peaks. Since the cell cycle duration in yeast is much shorter than that of mammalian cells (90 min vs. 24 hours, respectively), many consecutive cell cycles can be tracked in single yeast cells and consequently, spectral analysis can be beneficial in this case. We decided to overlay all tracks (even if partial) of the cell cycle to cancel out as many segmentation or measurements errors and then, a significant deviation from the average profile would indicate a cell-cycle dependent protein. For example, if we could track a specific cell only for 12 hours after it underwent mitosis and 10 hours before it underwent mitosis, we used both these partial tracks and overlay them on the cell-cycle profile. Both these partial tracks were normalized to the average cell cycle duration (21 hours) to overlay them on the normalized profile (See example in Fig S10). We considered clones as cell-cycle dependent if the calculated deviation had a low chance to occur by chance (using the bootstraing approach). We used the total protein in cell to calculate the cell-cycle dependent profiles, while other measures such as the median pixel or total protein/area can be used to assess the concentration of protein in a given frame. We found the median pixel in a cell to be a noisier measure for the protein level in our system (the average profile of the median pixel is shown in Fig S9 as well as the bootstrapping analysis ), but we added the score of deviation from the average profile when using the median pixel to our supplementary information.
